# Association of cetuximab with adverse pulmonary events in cancer patients: a comprehensive review

**DOI:** 10.1186/1756-9966-28-113

**Published:** 2009-08-14

**Authors:** Jeffrey B Hoag, Aimel Azizi, Timothy J Doherty, Jason Lu, Rudolph E Willis, Mark E Lund

**Affiliations:** 1 Cancer Treatment Centers of America, Eastern Regional Medical Center, Philadelphia, PA, USA; 2 Drexel University College of Medicine, Philadelphia, PA, USA

## Abstract

Compounds derived from biologic sources, or biologicals, are increasingly utilized as therapeutic agents in malignancy. Development of anti-cancer targeted therapies from biologics is increasingly being utilized. Cetuximab, a chimeric monoclonal antibody, is one such anti-cancer targeted therapeutic that has shown efficacy in quelling the rate of patient decline in colorectal, head/neck, and non-small cell lung cancer. However, due to the relatively recent addition of biologic compounds to the therapeutic arsenal, information related to adverse reactions is less well known than those seen in traditional chemotherapeutics. Dermatologic reactions have been demonstrated as the most frequent side effect cited during cetuximab therapy for malignancy; however, other effects may lead to greater morbidity. In general, pulmonary complications of therapeutics can lead to significant morbidity and mortality. The purpose of this review is to compile the various pulmonary side effects seen in patients treated with cetuximab for various malignancies, and to compare the incidence of these adverse reactions to standard therapies.

## Background

### Biological Therapies

Biologic therapies, or biologicals, are those produced or extracted from a biological source. Based upon the specific agent, biologicals have a myriad of activities and have been used to modulate immunity, increase blood cell production, inhibit tumor growth, and other effects [1]. Over the last 5 years, more than 20% of the compounds approved by United States regulatory authorities were biologics [2]. Despite this explosion in the availability of biologicals, surprisingly limited data exists regarding adverse events associated with their use.

Because these compounds are derived from biologic sources, they have the potential for significant immune activation. Although extensively reported in clinical trials, adverse events are rarely compiled in the medical literature. Giezen and coauthors examined adverse event reporting post-approval for biologicals and suggested that there was a need for increasing awareness to certain risks associated with the therapeutic use of biologicals [2].

### Cetuximab (Erbitux^®^)

One such biologic therapy used in the treatment of malignancy is cetuximab (Erbitux^®^, ImClone, Branchburg, NJ). Cetuximab is a chimeric monoclonal antibody with inhibitor effects on the epidermal growth factor receptor (EGFR). Cetuximab has been extensively studied and approved [3] for the treatment of metastatic colorectal cancer (MCRC) and squamous cell head/neck cancers (SCCHN), and growing data supports its use in the treatment of other malignancies including non-small cell lung cancer (NSCLC). Cetuximab has been evaluated in the setting of combination therapy or as a single agent in conventional therapy failures. Moreover, cetuximab has been studied for the treatment of various other malignancies including breast cancer and ovarian cancer, hepatocellular cancer, pancreatic cancer, and others.

Through binding to the extracellular domain of EGFR, cetuximab interrupts the signaling cascade resulting in inhibition of cell growth, induction of apoptosis, and decreased matrix metalloproteinase and vascular endothelial growth factor production [3]. EGFR, a member of the ErbB-1 family of receptors, is closely related structurally to other tyrosine kinase receptors including HER2/c-neu (ErbB-2), Her 3 (ErbB-3), and Her 4 (ErbB-4)[4]. Over expression or increased activity of EGFR as seen in some mutations can result malignancy [4].

Cetuximab efficacy has been studied as a single agent as well as in combination with other chemotherapeutic modalities. A randomized controlled clinical trial with 329 patients was conducted using cetuximab plus irinotecan or cetuximab alone in treatment of EGFR-expressing MCRC [3]. Cetuximab was shown to lengthen the time to disease progression by 4.2 months in the monotherapy arm and 5.7 months in combination arm. In patients with EGFR-positive NSCLC a phase II study by Rosell showed that combination cisplatin/vinorelbine plus cetuximab resulted in an overall response rate of 32%, compared to 20% with cisplatin/vinorelbine alone [5]. The continuing research of cetuximab is helping to determine which populations of patient will benefit most from Anti-EGFR therapy. Currently most evidence points towards the use of cetuximab in combination with other chemotherapeutic regimens as the best option for treatment in EGFR positive tumors.

Epidermal growth factor receptors are ubiquitous, thus potential for exuberant reactions including adverse events is high. Moreover, due to the diverse tissues expressing EGRF, adverse reactions manifest in many ways. Although dermatologic reactions represent the vast majority of adverse events, occurring in between 30-90% of patients depending on the severity and study examined [6,7], many other side effects occur with cetuximab therapy. Other adverse events increased above control groups included gastrointestinal complaints (19-59%) and headache (19%) [3]. Cextuximab infusion reactions took place in between 15 and 20% of subjects [3].

There have only been rare reports of pulmonary side effects with cetuximab. Interstitial lung disease was reported in 4 of 1,570 (0.25%) patients with advanced colorectal cancer [3]. There have also been reports of interstitial pneumonitis with non-cardiogenic pulmonary edema [8]. The use of cetuximab in combination regimens potentially clouds side effect profiles.

Pulmonary complications in the setting of chemotherapy lead to increased morbidity and severe reactions are associated with mortality. Cetuximab, like many other cancer therapies, has been demonstrated to cause a wide range of respiratory effects from mild dyspnea to a fatality due adverse pulmonary events. The purpose of this investigation is to compile a comprehensive list of pulmonary adverse events in the setting of therapy with cetuximab published in the literature in order to better characterize the true incidence of these reactions. A better understanding of the prevalence may help the clinician respond appropriately to specific symptom changes during the therapeutic window with a hope of improving patient care.

## Methods

We performed a MEDLINE™ search of the English language literature using the search terms: "cetuximab" or "Erbitux" with limits to include only human studies to develop a complete index of trials or reports. Inclusion criteria were clinical trials, meta-analyses, or randomized controlled trials that included the search terms and cited adverse events. The reference lists from each of these manuscripts were scanned to isolate articles not obtained in the MEDLINE^® ^search to complete our database. Studies were excluded if they did not list adverse events.

Data extracted from each report included number of patients, controls, type of cancer, coincident chemotherapy administration, and information regarding pulmonary complications. Pulmonary complications included the incidence of symptoms related to the respiratory system including dyspnea, cough, wheezing, pneumonia, hypoxemia, respiratory insufficiency/failure, pulmonary embolus, pleural effusion, and non-specific respiratory disorders. Incidences of these pulmonary complications were obtained from each study's control group if available and compared between the patients that received cetuximab and those who did not. Infusion reactions were treated as a separate complication to cetuximab and were not included in this analysis, although in many individuals, symptoms of shortness of breath and chest tightness may be encompassed by this type of reaction [9].

### Data Analysis and Statistics

Data is presented as the number of patients and percentage receiving the study medication as well as means (± SD) where appropriate. Comparisons between groups were made using Chi-Square or students t-test where appropriate, and statistical significance was set as p < 0.05.

## Results

Using our search criteria defined above, a total of 245 articles were obtained for review. From this complete group, 192 articles were excluded for not meeting inclusion criteria. Reasons for exclusion were: non-pulmonary focus (dermatologic side effects), duplicate patient populations, case reports not relevant to pulmonary side effects, focus on pharmacokinetics, omission of side effects, or non-cetuximab trials. A total of 53 studies (Table 1) met inclusion and were included in the analysis [5,8,10-60].

**Table 1:** Studies included in the analysis including first author, year of publication, type of trial and combined therapy, and pulmonary adverse reactions.

**Number**	**Ref:**	**Author**	**Year**	**Study Type**	**Cancer Type**	**Combined Agents**	**Pulmonary Reactions**
1	51	Vermorken	2008	Phase II	SCCHN	platinum	dyspnea, Pneumonia
2	29	Koo	2007	Phase II	Colorectal	FOLFIRI, irinotecan	dyspnea
3	8	Leard	2007	case	SCCHN		DAD
4	16	Burtness	2005	Phase III	SCCHN	cisplatin	dyspnea, hypoxia
5	15	Bourhis	2006	Phase I/II	SCCHN	cisplatin, carboplatin/5-fluorouracil	respiratory symptoms
6	12	Baselga	2005	Phase II	SCCHN	platinum	respiratory disorder
7	50	Vermorken	2007	review	SCCHN	cisplatinum, carboplatin	dyspnea, infusion rxn
8	20	Cunningham	2004	Prospective	Colorectal	irinotecan	dyspnea
9	53	Xiong	2004	Phase II	Pancreatic	gemcitabine	Pneumonia, Sepsis, PE, Pulm Insufficeincy
10	19	Chan	2005	Phase II	Nasopharyngeal	carboplatin	Pleural Effusion, Dyspnea, Pneumonia
11	40	Robert	2005	Phase I/II	NSCLC	gemcitibine, carboplatin	Pulmonary Embolism
12	23	Hanna	2006	Phase II	NSCLC		Dyspnea
13	54	Zhu	2007	Phase II	HCC		Cough
14	31	Machiels	2007	Phase I/II	Colorectal	capecitabine, ExBR	Pulmonary Embolism, Pulm Infetion
15	13	Bonner	2006	RCT	SCCHN	ExBR	cough, increased sputum
16	32	Martin-Martorell	2008	Phase II	Colorectal	irinotecan	none
17	28	Konner	2008	Phase II	Ovarian	carboplatin, paclitaxel	Dyspnea
18	56	Hughes	2008	Phase I/II	NSCLC	platinum, ExBR 64 Gy	dyspnea, pneumonitis, pulm embolism, pneumonia
19	11	Asnacios	2008	Phase II	HCC	oxaliplatin, gemcitabine	none
20	18	Cascinu	2008	Phase II	Pancreatic	cisplatin, gemcitabine	none
21	35	Paule	2007	Phase II	Cholangiocarcinoma	oxaliplatin, gemcitabine	none
22	47	Tabernero	2007	Phase II	Colorectal	oxaliplatin, 5-fluorouracil	Dyspnea
23	27	Jonker	2007	rescue	Colorectal	prev-oxal, irinotecan, flouropyrimidine	dyspnea
24	42	Safran	2008	phase II	Espohageal	carboplatin, paclitaxel, ExBR	Pneumonia
25	26	Ibrahim	2007	phase II	Colorectal	oxaliplatin, irinotecan	none
26	38	Pinto	2007	phase II	Gastric/GE	irinotecan, 5-fluorouracil	none
27	46	Souglakos	2007	phase II	Colorectal	oxaliplatin, capecitabine	none
28	30	Lenz	2006	phase II	Colorectal	prev-oxaloplatin, irinotecan, flouropyrimidine	none
29	25	Hofheinz	2006	phase I	Colorectal	capecitabine, irinotecan, ExBR	none
30	24	Herbst	2005	phase II	SCCHN	cisplatin	none
31	43	Saltz	2004	phase II	Colorectal	previous irinotecan	none
32	52	Vincenzi	2006	phase II	Colorectal	irinotecan	none
33	37	Pfister	2006	phase II	SCCHN	cisplatin, ExBR	pneumonia
34	39	Robert	2001	phase I	SCCHN	ExBR	none
35	48	Thienelt	2005	Phase I/II	NSCLC	carboplatin, paclitaxel	pulmonary embolism
36	34	Neyns	2008	Phase II	Colorectal	oxaloplatin or irinotecan	Interstitial pneumonitis
37	10	Arnold	2008	Phase Ib/II	Colorectal	oxaliplatin, 5-fluorouracil	None
38	45	Sobrero	2008	Phase III	Colorectal	irinotecan	None
39	44	Secord	2008	Phase II	Ovarian	carboplatin	Pulmonary rxn
40	14	Borner	2008	Phase II	Colorectal	oxaliplatin, capecitabine	none
41	21	Gamucci	2008	Phase II	Colorectal	irinotecan	none
42	49	Tol	2008	Phase III	Colorectal	capecitabine, Oxaliplatin + Bevacizumab	Pulmonary embolism, Respiratory Insuffiency
43	17	Butts	2007	Phase II	NSCLC	gemcitabine, Cisplatin, Carboplatin	dyspnea, Cough, Pneumonia
44	36	Pessino	2008	Phase II	Colorectal	none	none
45	5	Rosell	2008	Phase II	NSCLC	cisplatin, vinorelbine	resp symptoms
46	41	Rodel	2008	Phase I/II	Colorectal	capecitabine, ExBR, Oxaliplatin	none
47	33	Modi	2006	Phase I	Breast	paclitaxel	none
48	22	Gebbia	2006	retrospect rev	Colorectal	irinotecan	none
49	58	Pirker	2009	Phase III	NSCLC	cisplatin, vinorelbine	dyspnea, respiratory failure, pulmonary embolism
50	57	Belani	2008	Phase II	NSCLC	carboplatin, docetaxel	none
51	55	Gridelli	2009	Phase II	NSCLC	gemcitibine	pulmonary symptoms
52	59	Shin	2001	Phase I	SSCHN	Cisplatin	Shortness of Breath
53	60	Baselga	2000	Phase I	SSCHN/NSCLC	Cisplatin	Dyspnea

(Abbreviations: SCCHN - squamous cell cancer of the head and neck, NSCLC - non-small cell lung cancer, ExBR - external beam radiation)

The majority of clinical trials focused on the treatment of colorectal cancers with head/neck, lung, and hepatobiliary or pancreatic making up the next largest groups (Table 1). Similarly, most of the studies examined were completed as Phase I or II trials with the focus on refractory and metastatic disease (Table 2).

**Table 2:** Number and type of trials broken into groups according to cancer type.

Trial Type			Cancer Type			
	Colorectal	NSCLCa	Head-Neck	HB-Panc	Breast-Ov-Skin	TOTAL
Phase I/II	20	9*	10*	5	3	47
Phase III	2	1	1	0	0	4
Case Series/Review	1	0	1	0	1	3
TOTAL	23	10	12	5	4	53
						
First-Line	5	2	0	0	2	9
Refractory Disease	18	8*	12*	5	2	44

(NSCLCa - nonsmall cell lung cancer, HB-Panc - hepatobiliary or pancreatic, Breast-Ov-Skin - Breast or Ovarian or Cutaneous). * One study contained patients with either Head-Neck or Non-small cell lung cancer and is displayed in both groups.

A total of 7,411 patients were included in the 53 studies reviewed including 4,436 (59.8%) patients who received cetuximab either alone or in conjunction with other chemotherapeutic medications or radiation therapy. 2,596 (41.4%) patients were in the control groups from these investigations who received the same chemotherapy or radiation therapy without cetuximab (Table 3).

**Table 3:** Number of patients included by trial type.

Cancer Type	Studies Included	Total Patients	Number of Cetuximab	Cetuximab with Pulmonary Reaction	Number of Controls	Controls with Pulmonary Reaction
	n	n	n	n	n	n
Colorectal	23	3731	2227	76 (3.4)	1367	35 (2.6)
Head-Neck	10	1749	1004	173 (17.2)	516	104 (20.2)
Lung	10	1664	980	189 (19.6) †	671	82 (12.2)
Hepatobiliary/Pancreatic	5	209	167	9 (5.4)	42	0 (0.0)
Breast/Ovarian	3	78	78	10 (12.8)	0	0 (0.0)
Cutaneous	1	2	2	2 (100)	0	0 (0.0)
TOTAL:	52	7433	4458	459 (10.3) †	2596	221 (8.5)

Patients were grouped into those who received cetuximab, either alone or in combination with other therapeutics, and controls (those who did not receive cetuximab). † p < 0.05 compared to control group. * One study contained patients with either Head-Neck or Non-small cell lung cancer and is displayed in both groups.

### Pulmonary Reactions

A total of 459 patients (10.3%) in the cetuximab group had adverse pulmonary reactions compared to 221 (8.5%) who received standard, non-cetuximab therapy (p < 0.02). Studies focusing on colorectal cancer, lung cancer, and head-neck cancer had sufficient numbers in both the cetuximab and control groups to compare pulmonary complications; however, hepatobiliary, pancreatic, breast, ovarian, and cutaneous cancer studies lacked adequate numbers of control patients to compare these complications.

Colorectal cancer studies demonstrate a low rate of pulmonary complications overall with 3.41% incidence in the cetuximab group versus 2.56% in the control patients (p = NS). The most common side effect was dyspnea in these studies making up more than 90% of the adverse reactions.

Pulmonary adverse events were much more common, as would be expected in NSCLC trials with an incidence of 18.7% in the cetuximab group versus 12.2% in the control arms (p < 0.001). Similarly, dyspnea made up the majority of pulmonary adverse events (13.2% vs 9.2%, p < 0.02) with other significant differences occurring in the incidence of pneumonitis (1.1% versus 0.0%, p < 0.001) being worse in the cetuximab groups.

For head-neck cancer studies, the overall rates of pulmonary complications were similar between the cetuximab and control groups (17.9% versus 20.1%, p = NS), but favored the cetuximab group. Dyspnea was more common in the cetuximab group (8.7%) than the control group (5%, p < 0.02) in Head and Neck Cancer Trials. Conversely, there were fewer patients with increased sputum production (3.0% versus 6.6%, p < 0.01) and cough (4.5% versus 7.8%, p < 0.01) in the control group compared to the cetuximab group. From all studies, the difference in other pulmonary adverse events appears to be similar (Table 4).

**Table 4:** Combined pulmonary adverse events cited in clinical trials.

	Colorectal Cancer Cetuximab	Control	Non-Small Cell Lung Cancer Cetuximab	Control	Head-Neck Cancer Cetuximab	Control
	N (%)	N (%)	N (%)	N (%)	N (%)	N (%)
Dyspnea/RI	70 (3.1)	35 (2.6)	131 (13.4) †	62 (9.2)	87 (8.7) †	26 (5.0)
PE	3 (0.1)	0 (0.0)	32 (3.3)	16 (2.4)	0 (0.0)	0 (0.0)
Pneumonia	2 (0.1)	0 (0.0)	4 (0.4)	1 (1.2)	13 (1.4)	4 (0.8)
ILD	0 (0.0)	0 (0.0)	0 (0.0)	0 (0.0)	0 (0.0)	0 (0.0)
Cough	0 (0.0)	0 (0.0)	8 (3.4)	3 (3.6)	42 (4.5) †	40 (7.8)
Pneumonitis	1 (0.0)	0 (0.0)	17 (1.7) †	0 (0.0)	0 (0.0)	0 (0.0)
Pleural Effusion	0 (0.0)	0 (0.0)	0 (0.0)	0 (0.0)	3 (0.3)	0 (0.0)
Increased Sputum	0 (0.0)	0 (0.0)	0 (0.0)	0 (0.0)	28 (3.0) †	34 (6.6)
TOTAL:	76 (3.4)	35 (2.6)	192 (19.6) †	82 (12.2)	173 (17.9)	104 (20.2)

Patients were grouped into those who received cetuximab, either alone or in combination with other therapeutics, and controls (those who did not receive cetuximab). † p < 0.05 compared to control group.

## Discussion

Overall, cetuximab seems to increase the incidence of adverse pulmonary reactions compared to controls, although the absolute difference between groups is low (<2%). The severity of the pulmonary complications was not well described in most of the included studies, but did not increase mortality rates. To the contrary, if survival benefits were not demonstrated, almost universally, there was an increase in progression free survival or stability of malignancy in these trials. To this point, the difference between statistical significance and clinical significance should also be examined in relation to the pulmonary reactions. For all clinical trials except NSCLC, the differences in pulmonary adverse events between those treated with and without cetuximab are small. Dyspnea and cough, though increased in the cetuximab groups, did not appear to limit the therapeutic course.

The observation of increased pulmonary adverse events in patients with NSCLC when compared to controls was striking. Again, most of the adverse reactions in these patients were dyspnea or respiratory insufficiency, and were not noted to be treatment limiting. Although the mechanism for increased symptoms in patients with NSCLC is not well defined, it is not surprising that those with a site of action in the lung would suffer from exuberant local effects. Pneumonitis was seen in most patients (71%) treated with cetuximab in combination with radiation therapy for NSCLC, although there was no control group in this study for comparison [56]. These patients had advanced disease and were treated with a radiation dose of 64Gy to the lungs, which is well above the threshold for pneumonitis with radiation alone[61] As expected, treatment of head/neck cancers in these trials had high overall rates of pulmonary adverse events, although there were no significant differences between those who received cetuximab and those who did not.

Severe adverse reactions were not common in clinical trials using cetuximab. Interstitial lung disease, cited as a rare complication in the medication's package insert, was not described in the clinical trials included in this review with the exception of a case report of two post-lung transplantation patients treated with cetuximab for cutaneous malignancy. Obviously, there are likely confounding factors which may have predisposed this select population to the development of diffuse alveolar damage. For those described in the cetuximab package insert, interstitial lung disease was present before the institution of cetuximab therapy for malignancy. Arguably, the increase in pulmonary symptoms in these patients may have been more a manifestation of ILD progression than as an effect of the therapeutic. However, the presence of antecedent parenchymal lung disease may abrogate the utility of cetuximab in select patients. Pulmonary embolism, also considered a severe reaction, occurred in small numbers of patients in the groups analyzed herein.

An association between the presence of malignancy in the lung, regardless of primary origin, and pulmonary adverse events could not be determined from this investigation. Of the 43 non-lung cancer studies included in our series only 9 reported the location of metastatic disease. When combined with studies of lung cancer, 17% of this cohort reported direct pulmonary involvement of cancer. In those defining the sites of metastatic foci, the lungs were involved in 46.0 ± 10% of patients. Primary or metastatic involvement of the lung with any cancer could account for patients experiencing pulmonary adverse events when treated with Cetuximab. Unfortunately, a more clear relationship is limited by the presentation of the data in the original studies.

Our investigation suffers from several limitations which should be pointed out. First, it is a compilation of clinical trials, most of which are early phase, with limited numbers including control populations available for comparison of pulmonary adverse events. Most of the studies examined only cited positive adverse events, omitting negative responses to pulmonary symptom changes. This may lead to an over-estimation of the absolute incidence of pulmonary-specific complications. Conversely, transfusion reactions and sepsis which often include symptoms such as dyspnea or respiratory insufficiency were not included in the present analysis due to lack of a clear definition. There were significant differences in the duration of Cetuximab therapy before pulmonary complications were reported in the clinical trials, ranging from 1 week into therapy to more than several months. This also limits the generalizability of the summation data. Finally, although there appears to be an increase in the incidence of pulmonary adverse events with cetuximab therapy, there is no clearly defined causal relationship that can be proven as mechanistic understandings are lacking. Despite these limitations, we believe that this investigation adds to the sparse literature describing the pulmonary adverse events related to cetuximab therapy.

## Conclusion

Cetuximab (Erbitux^® ^ImClone, Branchburg, NJ) therapy, in combination or as monotherapy, is efficacious in the treatment of colorectal, head/neck, lung and possibly other cancers. Although there is an overall increase in the incidence of pulmonary adverse events with this treatment, there seems to be sparse evidence suggesting treatment limitations related to these complications. Particular attention should be given to cetuximab recipients with underlying parenchymal lung disease and those with NSCLC, in particular in conjunction with radiation therapy, as these groups may have more severe pulmonary reactions.

## Abbreviations

ILD: Interstitial lung disease; NSCLC: Non-small cell lung cancer; SCCHN: Squamous cell cancer/head and neck.

## Competing interests

The authors declare that they have no competing interests.

## Authors' contributions

JH conceived and designed the study and participated in writing. AA participated in data gathering, study screening, and study coordination. TD participated in data gathering, study screening, and study coordination. JL participated in statistical analysis of the study and study design. RW participated in study design and data analysis. ML performed oversight of study design, coordination, and writing. All authors read and approved the final manuscript.
